# Efficacy of Denosumab for Osteoporosis in Two Patients with Adult-Onset Still’s Disease—Denosumab Efficacy in Osteoporotic Still’s Disease Patients

**DOI:** 10.3390/jcm7040063

**Published:** 2018-03-22

**Authors:** Daiki Kumaki, Yukio Nakamura, Takako Suzuki, Hiroyuki Kato

**Affiliations:** 1 Department of Orthopaedic Surgery, Shinshu University School of Medicine, Asahi 3-1-1, Matsumoto 390-8621, Japan; 09m101023@gmail.com (D.K.); takako1119@shinshu-u.ac.jp (T.S.); hirokato@shinshu-u.ac.jp (H.K.); 2 Department of Orthopaedic Surgery, Showa Inan General Hospital, Akaho 3230, Komagane 399-4117, Japan

**Keywords:** adult-onset Still’s disease, bone mineral density, denosumab, osteoporosis

## Abstract

Adult-onset Still’s disease (AOSD) is an autoimmune inflammatory disorder. Glucocorticoids are often used for AOSD, which may induce complicating glucocorticoid-induced osteoporosis (GIO). An anti-resorption drug, denosumab, has recently been approved for osteoporosis treatment in Japan. However, the drug’s efficacy for GIO in AOSD is largely unknown. This retrospective, consecutive case series investigated two patients with GIO in AOSD to examine the effects of denosumab on bone metabolism. Bone turnover markers, and bone mineral density (BMD) of the lumbar 1–4 spine (L-BMD) and bilateral total hips (H-BMD) were followed for six months in a male patient and for twelve months in a female patient. No fractures or severe side effects, such as hypocalcemia, were observed during the observational period. Bone turnover markers were basically suppressed, and L-BMD and H-BMD were increased by denosumab in both patients. Our findings suggest that denosumab is a suitable candidate drug for GIO in AOSD.

## 1. Introduction

Adult-onset Still’s disease (AOSD) is an autoimmune inflammatory condition first described by Bywaters in 1972 [1]. The estimated prevalence of AOSD is currently 3.9 per 100,000 individuals. Analysis of 169 afflicted patients showed a mean onset age of 46 years [2]. Characteristic features of AOSD include rash, fever, peripheral joint, neck, and sacroiliac joint affections, biopsy findings, serology, and prognosis. Rheumatoid factor (RF) is typically absent or at low–normal titre, as in childhood, and antinuclear antibody (ANA) is negative [1,3]. Although AOSD onset has been suggested to accompany infection or malignant tumors, no causal relationships have been established to date and the etiology of AOSD remains unknown [4].

Non-steroidal anti-inflammatory drugs and glucocorticoids are primarily used for AOSD treatment, with recent reports of glucocorticoids and anti-rheumatoid disease-modifying drugs [5]. In either case, glucocorticoid-induced osteoporosis (GIO) is considered a major complication of AOSD. Although GIO treatment using bisphosphonates (BPs) as a first-line drug in AOSD patients had been described [6], both patients in the present study had refused BPs due to upper gastrointestinal intolerance. Accordingly, denosumab, an antibody against the human receptor activator of NF-κB ligand (RANKL) [7], was selected as an alternative agent. To the best of our knowledge, there have been no reports of the efficacy of denosumab for GIO in AOSD.

This study describes the clinical improvement of two cases of AOSD with GIO by denosumab therapy.

## 2. Case Presentation

### 2.1. Case 1

A 74-year-old man was treated for AOSD with osteoporosis. AOSD was diagnosed based on two major symptoms (arthralgia >2 weeks and fever >39 °C, intermittent >1 week) and five minor symptoms (sore throat, lymphadenopathy, abnormal liver function tests (LFT), negative ANA, and negative RF) in the department of internal medicine according to established criteria [8]. He had no remarkable medical history prior to the study. After AOSD diagnosis, oral prednisolone was prescribed at 20 mg per day for the first four months and then tapered by 1 mg per week thereafter. After eight months, the disease appeared to be in remission and a dose of 5 mg per day was continued for the next year. Due to the prednisolone treatment, however, he was diagnosed as having GIO based on the guidelines for the management and treatment of glucocorticoid-induced osteoporosis of the Japanese Society for Bone and Mineral Research (2014 update) [8]. Although he refused oral BPs due to gastroesophageal reflux disease symptoms, he was able to commence denosumab plus calcium (600 mg) and native vitamin D (400 IU) supplementation therapy. After three months of oral prednisolone at 5 mg per day, he began denosumab, injected subcutaneously every six months, and daily 0.75 μg eldecalcitol (ELD).

**Figure 1:**
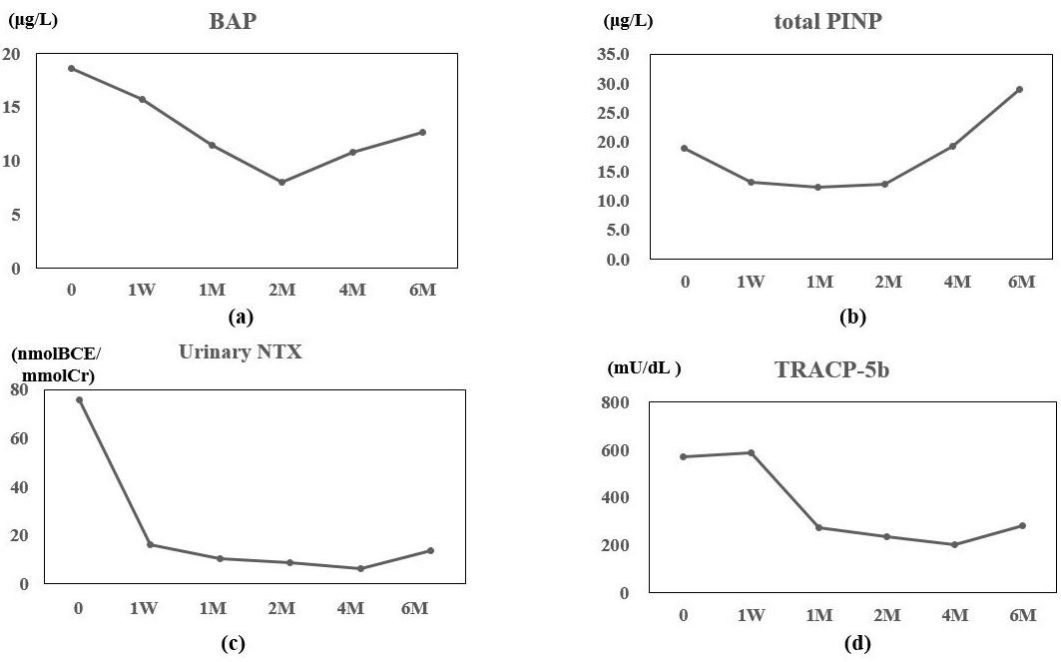

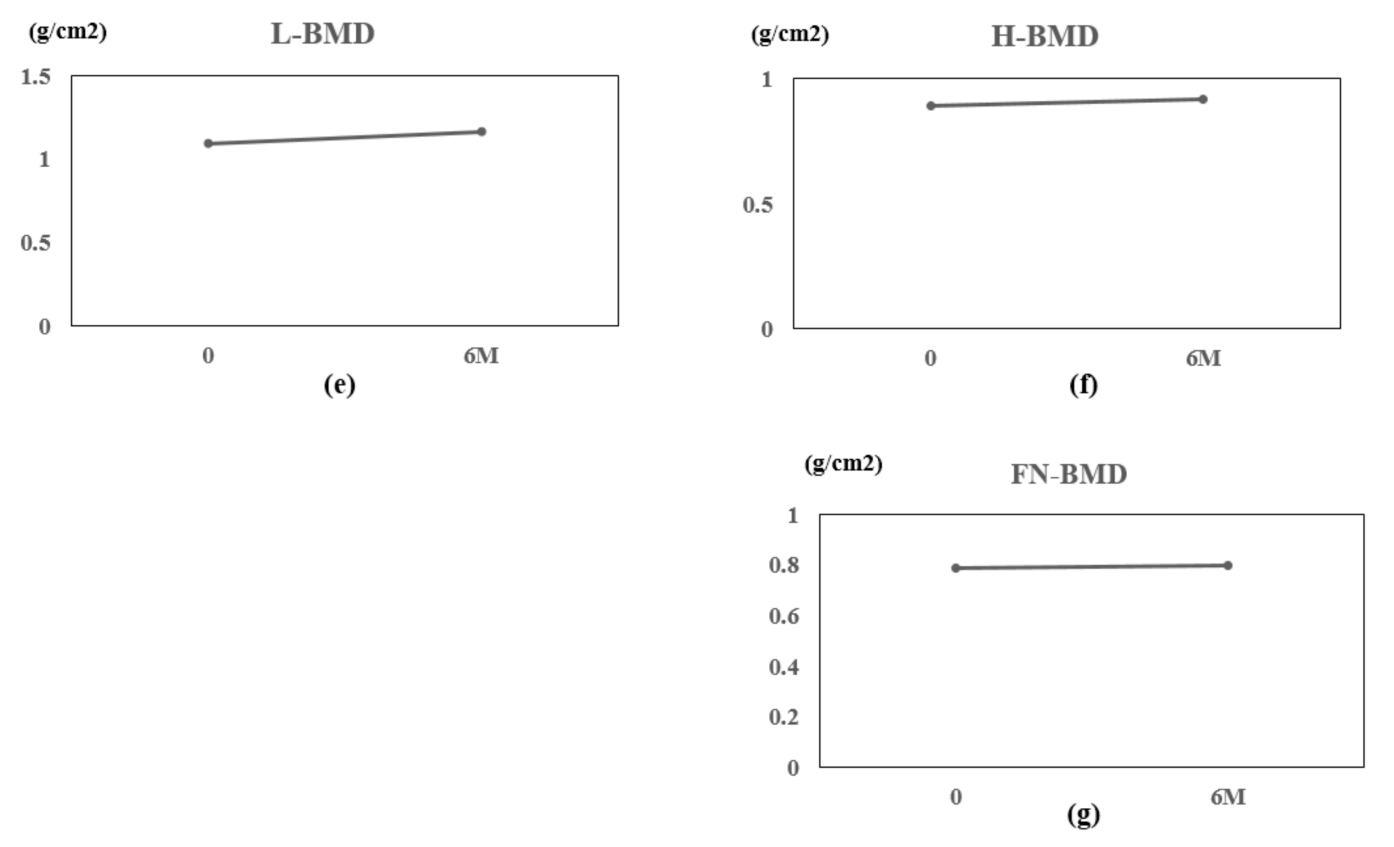
Patient 1 data: Value changes in bone alkaline phosphatase (BAP) (**a**), type I procollagen N-terminal propeptide (total P1NP) (**b**), urinary N-terminal telopeptide of type-I collagen (urinary NTX) (**c**), and tartrate-resistant acid phosphatase (TRACP)-5b (**d**), before and after one week (W) and one, two, four, and six months (M) of denosumab therapy. Value changes in lumbar 1–4 bone mineral density (L-BMD) (**e**), total hip BMD (H-BMD) (**f**), and femoral neck BMD (FN-BMD) (**g**) before and at 6 M of therapy.

Serum calcium and phosphorus values did not change remarkably (data not shown), while bone alkaline phosphatase (BAP) and total procollagen type I N-terminal propeptide (P1NP) values gradually decreased during two months of therapy, and then gradually increased (Figure 1a,b). Urinary N-terminal telopeptide of type-I collagen (NTX) values decreased from one week and remained low thereafter (Figure 1c). Serum tartrate-resistant acid phosphatase (TRACP)-5b values decreased afrom one monthand remained low thereafter (Figure 1d). Serum 1,25(OH)_2_D_3_ and whole parathyroid hormone (PTH) values transiently decreased at one month of therapy (data not shown). Lumbar 1–4 bone mineral density (L-BMD), total hip BMD (H-BMD), and femoral neck BMD (FN-BMD) increased gradually during therapy (Figure 1e–g).

The patient continues to receive prednisolone at 5 mg per day. Since he moved to another prefecture, his treatment was ceased at six months of treatment.

### 2.2. Case 2

Case 2 was a pre-menopausal 49-year-old woman with AOSD and osteoporosis. She was diagnosed in 2015 as having AOSD based on three major symptoms (arthralgia >2 weeks, fever >39 °C, intermittent >1 week, and characteristic rash) and five minor symptoms (sore throat, lymphadenopathy in neck, abnormal LFT, negative ANA, and negative RF) in the department of internal medicine according to published criteria [8]. She had no relevant medical history. After diagnosis, oral prednisolone was prescribed at 20 mg per day for the first four months, and then decreased by 1 mg per week thereafter. After five weeks of remission treatment, GIO was diagnosed as in Case 1 and therapy for osteoporosis was commenced. Subcutaneous daily teriparatide was given instead of bisphosphonate (BP) for six months due to upper gastrointestinal problems, which was switched to subcutaneous denosumab due to cost. She still takes 8 mg of glucocorticoids per day and has denosumab injections every six months.

**Figure 2:**
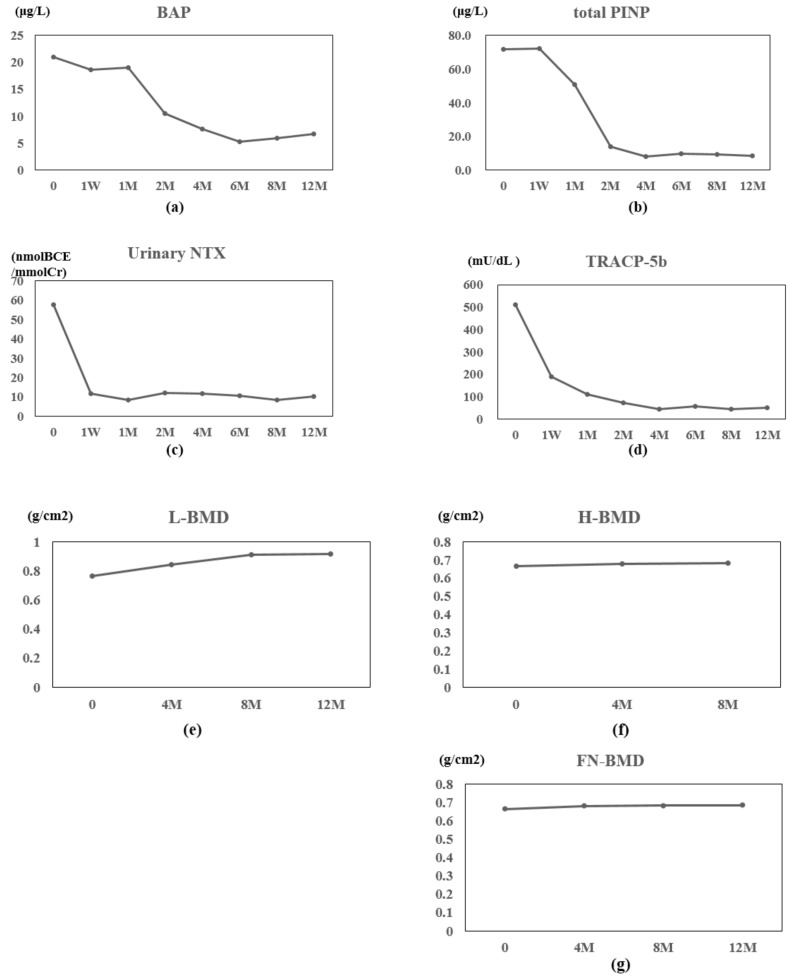
Patient 2 data: Value changes in bone alkaline phosphatase (BAP) (**a**), type I procollagen N-terminal propeptide (total P1NP) (**b**), urinary N-terminal telopeptide of type-I collagen (Urinary NTX) (**c**), and tartrate-resistant acid phosphatase (TRACP)-5b (**d**). before and at one week (W) and one, two, four, six, eight, and 12 months (M) of denosumab therapy. Value changes in lumbar 1–4 bone mineral density (L-BMD) (**e**), total hip BMD (H-BMD) (**f**), and femoral neck BMD (FN-BMD) (**g**) at the indicated time points.

Serum calcium and phosphorus values did not change substantially (data not shown). BAP and total P1NP gradually decreased after one month andone week of therapy, respectively, and stayed low thereafter (Figure 2a,b). TRACP-5b and urinary NTX were decreased at 1 week and remained low (Figure 2c,d). Serum 1,25(OH)_2_D_3_ transiently increased at one month and whole PTH transiently decreased at two months of therapy (data not shown). L-BMD, H-BMD, and FN-BMD increased gradually during treatment (Figure 2e–g).

In both patients, serum levels of BAP and total P1NP were measured as bone-formation markers using a chemiluminescent enzyme immunoassay and antibody radioimmunoassay. Serum levels of TRACP-5b and urinary levels of NTX (Osteomark^®^; Ostex International, Seattle, WA, USA) were assessed as markers of bone resorption using an enzyme-linked immunosorbent assay. Each marker was measured just before denosumab administration and at indicated times. BMD was measured using a dual-energy X-ray absorption fan-beam bone densitometer (Lunar Prodigy; GE Healthcare, Waukesha, WI, USA) at the lumbar 1–4 levels of the posteroanterior spine and bilateral total hips.

The study protocol was approved by the Ethics Committee of Shinshu University School of Medicine (Matsumoto, Japan) and Showa-Inan General Hospital (Komagane, Japan) and was carried out in accordance with the ethical standards set forth in the Declaration of Helsinki (2014 revision). Written consent for the publication of this case report was obtained from all patients prior to the start of the study.

## 3. Discussion

We herein report the successful improvement of GIO in two AOSD patients by denosumab therapy of six or 12 months that ameliorated BMD and bone turnover markers in both cases. No fractures or complications were recorded during the treatment period.

The pathogenesis of AOSD remains largely unknown, although several cytokines, such as interleukin-1 (IL-1), IL-6, IL-17, and tumor necrosis factor-alpha, may be important pro-inflammatory molecules, as reviewed by several research groups [9,10]. Serum IL-17 contributes to disease pathogenesis [9], and IL-1 is likely to be elevated in AOSD [10]. Karmakar et al. reported that IL-17 promoted bone erosion in a murine collagen-induced arthritis model by upregulating the expression of RANKL and RANK, thereby enhancing osteoclastogenesis [11]. Li et al. recently found that the IL-6 receptor monoclonal antibody, tocilizumab, exerted a favorable effect on recurrent AOSD [12]. These findings suggest that the RANK/RANKL pathway represents a pathogenic mechanism of bone erosion and/or destruction in arthritic diseases, including AOSD. We and others have found denosumab to be useful for GIO patients [8,12,13,14,15]. Although BPs are first-line drugs for GIO, the current patients exhibited upper gastrointestinal issues that precluded BP use. Denosumab inhibits osteoclastogenesis via the RANK/RANKL pathway, thus, we switched from daily teriparatide for six months to denosumab for one year in the female patient and gave denosumab for six months to the male patient for GIO in AOSD.

Vitamin D is required for optimal BMD increase during denosumab treatment [16]. ELD, an analog of active vitamin D3, could improve BMD and reduce the risk of osteoporotic fractures [17]. We previously reported that a combination therapy with denosumab and ELD ameliorated FN-BMD more effectively than did denosumab plus native vitamin D [18]. In this study, a 0.9% increase of FN-BMD with ELD at six months in Case 1 and a 2.4% gain in FN-BMD without ELD at four months in Case 2 were observed during denosumab therapy. Nonetheless, a combination therapy of anti-resorption drugs and vitamin D, such as ELD, may produce better effects on bone metabolism and BMD, as reported previously [17,18].

The limitations of the current study include the lack of a control group, the small sample size, different genders, and retrospective design. Nevertheless, our series provided evidence of a favorable response in BMD after six to 12 months of denosumab treatment in the rare disease AOSD.

In conclusion, BMD and bone turnover markers were improved by denosumab without fractures or adverse events in two AOSD patients with complicating GIO. Denosumab therefore represents a suitable option for GIO treatment in AOSD.

## Supplementary Materials

The following are available online at http://www.mdpi.com/2077-0383/7/4/63/s1. Table S1. Patient characteristics and lumbar and total hip bone mineral density prior to denosumab therapy.

## References

[1] Bywaters E.G. (1971). Still’s disease in the adult. Ann. Rheum. Dis..

[2] Asanuma Y.F., Mimura T., Tsuboi H., Noma H., Miyoshi F., Yamamoto K., Sumida T. (2015). Nationwide epidemiological survey of 169 patients with adult Still’s disease in Japan. Mod. Rheumatol..

[3] Magadur-Joly G., Billaud E., Barrier J.H., Pennec Y.L., Masson C., Renou P., Prost A. (1995). Epidemiology of adult Still’s disease: Estimate of the incidence by a retrospective study in west France. Ann. Rheum. Dis..

[4] Cagatay Y., Gul A., Cagatay A., Kamali S., Karadeniz A., Inanc M., Ocal L., Aral O., Konice M. (2009). Adult-onset Still’s disease. Int. J. Clin. Pract..

[5] Kádár J., Petrovicz E. (2004). Adult-onset Still’s disease. Best Pract. Res. Clin. Rheumatol..

[6] Mok C.C., Tong K.H., To C.H., Siu Y.P., Ma K.M. (2008). Risedronate for prevention of bone mineral density loss in patients receiving high-dose glucocorticoids: A randomized double-blind placebo-controlled trial. Osteoporos. Int..

[7] McClung M.R., Lewiecki E.M., Cohen S.B., Bolognese M.A., Woodson G.C., Moffett A.H., Peacock M., Miller P.D., Lederman S.N., Chesnut C.H. (2006). Denosumab in postmenopausal women with low bone mineral density. N. Engl. J. Med..

[8] Suzuki Y., Nawata H., Soen S., Fujiwara S., Nakayama H., Tanaka I., Ozono K., Sagawa A., Takayanagi R., Tanaka H. (2014). Guidelines on the management and treatment of glucocorticoid-induced osteoporosis of the Japanese Society for Bone and Mineral Research: 2014 update. J. Bone Miner. Metab..

[9] Efthimiou P., Georgy S. (2006). Pathogenesis and management of adult-onset Still’s disease. Semin. Arthritis Rheum..

[10] Yamaguchi M., Ohta A., Tsunematsu T., Kasukawa R., Mizushima Y., Kashiwagi H., Kashiwazaki S., Tanimoto K., Matsumoto Y., Ota T. (1992). Preliminary criteria for classification of adult Still’s disease. J. Rheumatol..

[11] Karmakar S., Kay J., Gravallese E.M. (2010). Bone damage in rheumatoid arthritis: Mechanistic insights and approaches to prevention. Rheum. Dis. Clin. N. Am..

[12] Li T., Gu L., Wang X., Guo L., Shi H., Yang C., Chen S. (2017). A pilot study on Tocilizumab for treating refractory adult-onset Still’s disease. Sci. Rep..

[13] Ishiguro S., Ito K., Nakagawa S., Hataji O., Sudo A. (2017). The clinical benefits of denosumab for prophylaxis of steroid-induced osteoporosis in patients with pulmonary disease. Arch. Osteoporos..

[14] Sawamura M., Komatsuda A., Togashi M., Wakui H., Takahashi N. (2017). Effects of denosumab on bone metabolic markers and bone mineral density in patients treated with glucocorticoids. Intern. Med..

[15] Suzuki T., Nakamura Y., Kato H. (2018). Significant improvement of bone mineral density by denosumab without bisphosphonate pre-treatment in glucocorticoid-induced osteoporosis. Mod. Rheumatol..

[16] Matsumoto T., Ito M., Hayashi Y., Hirota T., Tanigawara Y., Sone T., Fukunaga M., Shiraki M., Nakamura T. (2011). A new active vitamin D_3_ analog, eldecalcitol, prevents the risk of osteoporotic fractures—A randomized, active comparator, double-blind study. Bone.

[17] Mukaiyama K., Uchiyama S., Nakamura Y., Ikegami S., Taguchi A., Kamimura M., Kato H. (2015). Eldecalcitol, in combination with bisphosphonate, is effective for treatment of Japanese osteoporotic patients. Tohoku J. Exp. Med..

[18] Suzuki T., Nakamura Y., Tanaka M., Kamimura M., Ikegami S., Uchiyama S., Kato H. (2017). Comparison of the effects of denosumab with either active vitamin D or native vitamin D on bone mineral density and bone turnover markers in postmenopausal osteoporosis. Mod. Rheumatol..

