# Supplementary material for: Efficacy of Denosumab for Osteoporosis in Two Patients with Adult-Onset Still’s Disease—Denosumab Efficacy in Osteoporotic Still’s Disease Patients
Source: J Clin Med. 2018 Mar 22;7(4):63. doi: 10.3390/jcm7040063 (PMC5920437; doi:10.3390/jcm7040063)
Supplement: Supplementary file 1 [file jcm-07-00063-s001.pdf]

Journal of
*Clinical Medicine*

## Supplementary

**Table S1.** Patient characteristics and lumbar and total hip bone mineral density prior to denosumab therapy.

| Characteristic | Case 1 | Case 2 |
|---|---|---|
| Age (years) | 73 | 48 |
| Gender | Male | Female |
| Ethnicity | Japanese | Japanese |
| Glucocorticoids (mg/day) | 20 | 20 |
| Lumbar1-4 BMD (L-BMD) ($g/cm^2$) | 1.091 | 0.766 |
| T score of L-BMD | -0.7 | -2.9 |
| Total hip BMD (H-BMD) ($g/cm^2$) | 0.894 | 0.664 |
| T score of H-BMD | -0.4 | -2.3 |
